# Temperature modulates dominance of a superinfecting Arctic virus in its unicellular algal host

**DOI:** 10.1093/ismejo/wrae161

**Published:** 2024-08-22

**Authors:** Claudia Meyer, Victoria L N Jackson, Keith Harrison, Ioanna Fouskari, Henk Bolhuis, Yael A Artzy-Randrup, Jef Huisman, Adam Monier, Corina P D Brussaard

**Affiliations:** Department of Marine Microbiology and Biogeochemistry, NIOZ Royal Netherlands Institute for Sea Research, PO Box 59, 1790AB Den Burg, Texel, The Netherlands; Department of Freshwater and Marine Ecology, Institute for Biodiversity and Ecosystem Dynamics (IBED), University of Amsterdam, PO Box 94240, 1090 GE Amsterdam, The Netherlands; Living Systems Institute, University of Exeter, Exeter, Devon EX4 4QD, United Kingdom; Living Systems Institute, University of Exeter, Exeter, Devon EX4 4QD, United Kingdom; Department of Marine Microbiology and Biogeochemistry, NIOZ Royal Netherlands Institute for Sea Research, PO Box 59, 1790AB Den Burg, Texel, The Netherlands; Department of Marine Microbiology and Biogeochemistry, NIOZ Royal Netherlands Institute for Sea Research, PO Box 59, 1790AB Den Burg, Texel, The Netherlands; Department of Theoretical and Computational Ecology, Institute for Biodiversity and Ecosystem Dynamics (IBED), University of Amsterdam, PO Box 94240, 1090 GE Amsterdam, The Netherlands; Department of Freshwater and Marine Ecology, Institute for Biodiversity and Ecosystem Dynamics (IBED), University of Amsterdam, PO Box 94240, 1090 GE Amsterdam, The Netherlands; Living Systems Institute, University of Exeter, Exeter, Devon EX4 4QD, United Kingdom; Department of Marine Microbiology and Biogeochemistry, NIOZ Royal Netherlands Institute for Sea Research, PO Box 59, 1790AB Den Burg, Texel, The Netherlands; Department of Freshwater and Marine Ecology, Institute for Biodiversity and Ecosystem Dynamics (IBED), University of Amsterdam, PO Box 94240, 1090 GE Amsterdam, The Netherlands

**Keywords:** virus–virus interactions, coinfection, marine phytoplankton, Arctic Ocean, transcriptomics, viral genome

## Abstract

Complex virus–virus interactions can arise when multiple viruses coinfect the same host, impacting infection outcomes with broader ecological and evolutionary significance for viruses and host. Yet, our knowledge regarding virus competition is still limited, especially for single-celled eukaryotic host-virus systems. Here, we report on mutual interference of two dsDNA viruses, MpoV-45T and MpoV-46T, competing for their Arctic algal host *Micromonas polaris*. Both viruses affected each other’s gene expression and displayed reduced genome replication during coinfection. MpoV-45T was the dominant virus, likely due to interference in the DNA replication of is competitor. Even when its coinfection was delayed, the dominant virus still prevailed while genome production of the other virus was strongly suppressed. This contrasts with typical superinfection exclusion, where the primary infection prevents secondary infection by other viruses. Higher temperature made the suppressed virus a stronger competitor, signifying that global warming is likely to alter virus–virus interactions in Arctic waters.

## Introduction

Coinfection by multiple viruses is widespread in nature, and can occur at different levels, from infecting the same multicellular host to infecting the same individual host cell [1–6]. Resultant virus–virus interactions include virus interference, where the presence of one virus species or strain within the same host affects the outcome of infection and/or replication of the other virus [1, 5, 6]. Superinfection commonly leads to the preexisting viral infection restricting a secondary infection, with only the first virus being able to replicate [5–9]. Still, the secondary virus may also interfere with the replication of the virus already present, causing a reduction in viral yield [7]. Single-celled hosts, thus far mainly bacteria, act as valuable model systems for coinfection studies [10, 11], allowing for controlled one-step growth experiments, easy manipulation of experimental conditions, and improved reproducibility. However, single-celled eukaryotes have hardly been used as coinfection models, despite their global importance. Underlying mechanisms are unclear, and a better understanding of the occurrence and ecological relevance of virus–virus interactions in eukaryotes is needed.

Prokaryotic and eukaryotic unicellular algae (phytoplankton) are the dominant marine primary producers, sequestering vast amounts of carbon dioxide and supplying around 50% of the world’s oxygen [12]. Phytoplankton growth depends on several environmental factors, such as nutrients, light, and temperature, and is very sensitive to global change [13, 14]. For example, the loss of ice cover across vast areas of the Arctic Ocean has led to a longer growing season and increased open-water habitat for phytoplankton growth [15]. As a result, the annual net primary production of the Arctic Ocean has strongly increased in recent decades [16], and ocean-climate models predict that it will continue to rise in the future [17].

Like all life on Earth, phytoplankton are susceptible to viral infections [18]. Viral lysis of phytoplankton is an important mortality factor with profound consequences for the flow of energy and matter [19–22]. Moreover, viral infection impacts host diversity through selective infection and emerging host resistance [23–26]. Multiple virus strains may be able to infect the same phytoplankton host strain [27–31] and compete for successful infection and virus replication. Studies focusing on competition between phytoplankton viruses are, however, rare (except for two studies [8, 9] investigating freshwater *Chlorella* viruses, and one [32] studying viruses coinfecting the marine coccolithophore *Emiliania huxleyi*). This implies that our current understanding of marine viruses competing for the same phytoplankton host is still in its infancy, whereas effects of temperature and other environmental factors on competition between phytoplankton viruses are unknown.

Here, we investigate the competition dynamics of two viruses infecting the same eukaryotic phytoplankton host, the unicellular alga *Micromonas polaris* which typically dominates the picophytoplankton fraction in the Arctic Ocean [33–35]. Members of the ubiquitous genus *Micromonas* are known to be readily infected with dsDNA viruses belonging to the Phycodnaviridae [30, 31, 36, 37]. Currently, four virus strains infecting *M. polaris* are held in culture (MpoV-44 T, −45 T, −46 T, and -47 T [31]). We chose MpoV-45T and MpoV-46T because they are genomically different (this study) but have comparable virus replication cycles (e.g., similar latent period). Using one-step infection experiments, we added either MpoV-45T only, MpoV-46T only, or both MpoV-45T and MpoV-46T (dual infection) to *M. polaris*. We tested the following alternative hypotheses: dual infection does not affect virus replication (non-interference, H0); leads to one-sided interference (replication of only one of the viruses is affected, H1); or mutual interference (replication of both viruses is affected, H2). We show that dual infection resulted in significantly altered gene expression as well as reduced genome replication for both viruses. MpoV-46T was repressed considerably, even in superinfection treatments with delayed introduction of MpoV-45T. The dominance of MpoV-45T was overturned when growth temperature was increased from 3°C to 7°C, underlining the importance of temperature as a modulating factor for virus–virus interactions.

## Materials and methods

### Culturing conditions

The Arctic picoeukaryotic photoautotroph *Micromonas polaris* strain RCC2258 (Roscoff Culture Collection) was cultured at 3°C and 7°C under 75 μmol quanta m^−2^ s^−1^ (40FL Panasonic 40SS-ENW/37 lamps) at a 16:8 h L:D cycle. Algal cultures were long-term acclimated to their respective growth conditions. Cultures were kept in exponential growth phase (maximum growth rate 0.39 and 0.56 d^−1^ at 3°C and 7°C) by regular transfer to fresh Mix-TX medium [31].

The *M. polaris*-specific dsDNA prasinoviruses (Phycodnaviridae) MpoV-45T and MpoV-46T [31] (NIOZ culture collection) have a particle size of ~120 nm, possess a lipid membrane, have comparable estimated genome size, infection dynamics, latent period, and burst size [31]. The viruses were maintained by regular transfer of lysate to exponentially growing *M. polaris* RCC2258 (10% v/v).

### Experimental setup competition experiments

For the one-step infection experiments, exponentially growing *M. polaris* host cultures (target concentration of 2 × 10^5^ cells ml^−1^) were infected with freshly produced MpoV-45T, or MpoV-46T lysate (single infection treatment), or with both viruses (dual treatment). The virus to algal host cell ratio (v:a) at the start of the experiments was between 7 and 12, and the percentage of infective viruses (obtained by endpoint dilution [31]) was around 40% and 60% for MpoV-45T and MpoV-46T for both 3°C and 7°C. The viruses were added to the host around 3 h into the light cycle (lights on at 8 a.m.), except for the experiments where MpoV-45T was added with 6, 12, 18 and 24 h delay after the addition of MpoV-46T. Experiments were performed in triplicate. Experimental results shown are from two separate experiments at 3°C with good replication (Fig. S1) and one experiment at 7°C. Samples were taken directly upon infection (T0) and subsequently at regular intervals for up to 120 h post infection (p.i.). For flow cytometric analysis of virus abundance, 1 ml sample was fixed with 25% glutaraldehyde (EM-grade, Sigma-Aldrich, St. Louis, MO, USA) to a final concentration of 0.5% [38]. For flow cytometric enumeration of the phytoplankton host, 1 ml sample was fixed with a formaldehyde: hexamine solution (18% v/v: 10% wt/v) to a final concentration of 0.5% formaldehyde. All fixed samples were incubated at 4°C for 15 min, flash frozen in liquid nitrogen and stored at −80°C until analysis. For quantitative polymerase chain reaction (qPCR) analysis, 1 ml samples were immediately stored at −80°C until further processing.

### Flow cytometry

Fixed phytoplankton were enumerated using a Becton Dickinson Accuri C6 flow cytometer equipped with a 488 nm argon laser, with the trigger set to chlorophyll red autofluorescence [39]. The fixed virus samples were enumerated using a BD FACSCanto flow cytometer equipped with a with a 488 nm argon laser, with the trigger on green fluorescence [38]. In short, frozen samples were thawed and diluted in TE buffer (pH = 8.2, 10 mM Tris–HCl, 1 mM EDTA [40]) filtered through 0.2 μm Minisart NML cellulose acetate filters (Sartorius AG, Göttingen, Germany). Samples were subsequently stained with the nucleic acid-specific green fluorescent dye SYBR-green I (10^−5^ dilution of commercial stock, ThermoFisher Scientific, Waltham, EUA) at 80°C in the dark for 10 min. Afterwards, samples were left to cool at room temperature for at least 5 min before enumeration. FCS express 5 (De Novo Software, Pasadena, CA, USA) as well as BD Accuri C6 plus and BD FACS Diva software (BD Biosciences, Franklin Lakes, NJ, USA) was used to analyse the FCM data. For phytoplankton, gating was performed on red chlorophyll autofluorescence vs. side scatter, and for viruses on green fluorescence vs. side scatter. Discriminating phytoplankton cells based on chlorophyll fluorescence may result in slight overestimation of the timing of host cell lysis. Progeny viruses of *Micromonas pusilla* seem to release in a number of small expulsions by localized rupture of the host membrane [41] and live/dead staining indicates *Micromonas* cell membrane is compromised at the time of viral progeny release [42], while chlorophyll fluorescence of the host measured by flow cytometry might still be intact at this timepoint [43].

### MpoV-specific qPCR

Primers used to target the viral DNA polymerase B gene (*polB*) for MpoV-45T were F1 (ACTACGAAACCTTCGATTTG) and R1 (TCATACTGATCTTATAGGCG) at an annealing temperature of 59.1°C, and for MpoV-46T F1 (CGTGATGGATGAGAGACGC) and R1 (GAAACTGTTTGAGCTCCGCT) at an annealing temperature of 54.1°C. To assess primer efficiency and produce calibration curves, dilution series of purified PCR product of the respective primers was prepared. To obtain purified PCR product, fresh viral lysates were used as template. Amplicons were produced in 25 μl total volume using standard PCR conditions: 1.0 U BiothermPlus DNA Polymerase, 1x Biotherm Reaction buffer containing 1.5 mM MgCl_2_ (GeneCraft UK Products Semiramis Genetics Ltd., Manchester, UK), 0.8 μM of each primer, 0.25 mM of each dNTP, 0.2 mg L^−1^ of BSA. A BioRad T100 Thermocycler (Bio-Rad Laboratories, Inc., Hercules, USA) was used with the following program: initial denaturation for 4 min at 94°C followed by 40 cycles of 30 s denaturation at 94°C, 30 s annealing at the designated annealing temperature and 1 min elongation at 72°C, and a final elongation step at 72°C for 7 min. After amplification, the PCR product was loaded on a 2% agarose gel in 1x TAE buffer (40 mM Tris, 21 mM acetic acid, 1 mM EDTA) and stained with 0.25 X SYBR Safe (Invitrogen, Carlsbad, USA) and run at 80 V for around 85 min. The product bands were cut out under blue light and the amplicon was purified using the QIAquick Gel Extraction Kit (Qiagen Sciences Inc, Germantown, USA). The extracted product was measured using the Qubit HS DNA assay on a Qubit 3.0 fluorometer (Invitrogen, Carlsbad, USA). Dilution series were set up with a targeted concentration range of 10^9^ to 10 copies per μl in Tris buffer, immediately aliquoted, and stored at −20°C. To get more accurate qPCR measurements, the 10^9^ dilution was measured again on the Qubit 3.0 fluorometer (Invitrogen, Carlsbad, USA).

The frozen qPCR samples from the experiments were thawed at 4°C and 1 ml of sample was diluted with 4 ml of cold ultrapure water (18.2 Ω cm^−2^). Samples were then 3 times sonicated using a MSE Soniprep 150 Ultrasonic disintegrator (MSE Ltd., London, United Kingdom) at an amplitude of ~8 for 10 s with a break of 30 s in between. Samples were kept on ice before, during, and after sonication. Samples were then aliquoted and stored at −80°C until use. QPCR analysis was performed using the Bio-Rad CFX96 Touch Real-Time PCR Detection System (Bio-Rad Laboratories, Inc., Hercules, USA) with an initial denaturation at 94°C followed by 40 cycles of 30 s denaturation at 94°C, 30 s annealing at the designated annealing temperature and 1 min elongation at 72°C. After amplification, a melting curve from 65°C until 95°C, with 0.5°C increments, was performed to assess product length. Each qPCR was carried out in 25 μl (total volume) comprising 1.0 U BiothermPlus DNA Polymerase, 1x Biotherm Reaction buffer (containing 1.5 mM MgCl2), 0.8 μM of each primer, 0.25 mM of each dNTP, 0.4 h L^−1^ of BSA, 200x SYBR Green (Invitrogen, Carlsbad, USA), and 5 μl of sonicated sample. For the 7°C experiment, qPCRs were prepared using 5 μl of sonicated sample added to a 25 μl (total volume) PCR containing 1X AccuStar II PCR ToughMix (Quantabio, LLC. Beverly, MA, USA), 0.8 μM of each primer and 200x SYBR Green (Invitrogen, Carlsbad, USA). Non-template controls containing ultrapure water instead of sample, as well as off-target controls containing samples with the non-targeted viruses were included to control for primer specificity. QPCR assays for sample analysis included a calibration curve based on triplicates of the dilution series for the first plate, subsequent plates were normalized to the first plate using internal standards of samples collected during the respective experiments. Efficiencies for the qPCRs were 99.5 ± 10.9% for the MpoV-45T primer pair and 101.1 ± 7.4% for the MpoV-46T primer pair (n = 3, separate qPCR assays).

### Experimental data analysis and statistics

Latent period was defined as the period in which viral particles (based on flow cytometry) increased by at least 10% compared to the previous timepoint. Viral particle burst size was determined by dividing the number of viruses produced (maximum increase in flow cytometric virus counts) by the number of lysed host cells (maximum decline in algal counts). Similarly, to calculate viral genome burst size, the number of *polB* copies produced was divided by the number of lysed host cells. To compare different primer pairs and experiments, *polB* production was then expressed as a percentage of the respective single treatment. Maximum viral particle and genome production rates were calculated by fitting a linear regression over the period with the steepest slope of increase (Fig. S2). For qPCR data analysis, results for each replicate were min-max normalized to the average maxima and minima of the respective single virus treatment for better comparability between different sets of qPCRs (different primer pairs as well as separate experiments). Maximum production rates for *polB* were calculated based on normalized data. For statistical analysis R version 4.2.1 was used. Statistical differences were calculated using either a t-test or ANOVA followed by a Tukey HSD (Tukey multiple comparison of means) test. Applicability of parametric tests was tested using a Levene’s test for homogeneity of variances. If variances were not homogenous, a Wilcoxon rank sum test or a Kruskal-Wallis rank sum test followed by a Dunn’s test of multiple comparisons were performed. Statistical tests for viral burst size and production rates were performed on raw data. For viral genome production rates, tests between single treatments and dual treatments within one data set were performed on raw data. Tests between dual treatments of different experiments were performed on normalized data. Viral genome production rates were always based on normalized data.

### Viral DNA extraction

For virus sequencing (not previously sequenced), fresh virus lysate was prepared as described above. Fully lysed lysate was filtered using 0.22 μm MiniSart PES polyethersulfone filters (Sartorius AG, Göttingen, Germany). Lysates were kept at 4°C until further processing. Concentration of the filtered lysate was carried out by tangential flow filtration (100 000 MWCO PES membrane VivaFlow 200 module; Sartorius, Göttingen, Germany) prior to virus precipitation and DNA extraction. Lysates had to be concentrated to maximize DNA yield as preliminary DNA extractions yielded concentrations too low for Nanopore sequencing. Test extractions on unconcentrated lysates yielded higher DNA concentrations for MpoV-45T than MpoV-46T, so a higher concentration factor was used for MpoV-46T than MpoV-45T. For MpoV-45T, 250 ml filtered lysate were concentrated to 200 ml, and for MpoV-46T, 400 ml lysate were concentrated to 200 ml. Viral particles were precipitated as follows: PEG8000 (10% w/v) and NaCl (6.5% w/v) were dissolved in concentrated lysates and incubated on ice at 4°C overnight, followed by a centrifugation step for 1 h at 12 000 × *g*. The tubes were carefully removed from the centrifuge so as not to disturb the virus pellets. The supernatant was poured out and empty tubes placed upside down on a paper towel to drain any remaining liquid. A P1000 pipette was used to vigorously wash the pellet off the inside of the tubes with 1 ml SM buffer, which was collected in a 2 ml microcentrifuge tube. This step was repeated for each lysate, ensuring the pipette was wiped clean between each tube. DNA was extracted using the Wizard DNA Clean-Up System (Promega, Madison, WI, USA). One ml of resin (shaken before use) was added to each 1 ml of previously collected SM buffer and mixed by inversion. The mixture was then pushed through the Wizard columns using a syringe (1 ml at a time), followed by 2 ml 80% isopropanol. The columns were placed securely back in the original 2 ml microcentrifuge tubes and centrifuged for 2 min at 10 000 × *g* to remove excess isopropanol. Columns were placed in 1.5 ml Eppendorf LoBind tubes and 102 μl nuclease-free water (preheated to 65°C) was added to the center of the column and incubated for 2 min. A final centrifugation step was carried out for 30 s at 10 000 × *g* to collect the eluted DNA from the columns, and 2 μl was used for quantification by Qubit fluorometer (Invitrogen, Waltham, MA, USA) using the 1 X dsDNA HS assay.

A subsequent DNA concentration step was carried out: 3 M pH 5.2 sodium acetate was added to the DNA solution to give a final concentration of 0.3 M sodium acetate. Then, 0.7 volumes of room temperature isopropanol were added, and the tubes were stored at −80°C for 20 min to precipitate the DNA. Samples were then centrifuged at 15 000 × *g* for 30 min at 4°C to pellet the DNA. The supernatant was carefully removed, and the pellet washed with 500 μl 70% ethanol at room temperature. Thorough mixing by flicking and inverting the tube ensures removal of residual isopropanol. This was followed by another centrifugation step at 15 000 × *g* for 30 min at 4°C. Pellets were visually identified and the ethanol was carefully poured out. The tubes were inverted and placed on a paper towel to drain, and a pipette was used where required to remove residual ethanol. Pellets were then air-dried for 10–20 min before being resuspended in 27 μl nuclease-free water and incubated at 4°C overnight. Two μl were used for quantification by Qubit fluorometer using the 1 X dsDNA HS assay. For MpoV-45T, 6.3 ng μl^−1^ extracted DNA was concentrated to 10.5 ng μl^−1^. For MpoV-46T, 10.5 ng ^μ-1L^ was concentrated to 14.4 ng μl^−1^.

### DNA library preparation and Nanopore sequencing

DNA libraries were prepared for nanopore sequencing using the Ligation Sequencing Kit (SQK-LSK109; ONT, Oxford, UK) following the manufacturer’s instructions and ligation sequencing gDNA protocol GDE_9063_v109_revAK_14Aug2019. A 30 μl reaction volume was used for the DNA repair and end-prep step, with 24 μl viral DNA being used and DNA CS omitted. The reaction was incubated for 20 min at 20°C followed by 5 min at 65°C on a thermocycler. A 1 × AMPure XP bead clean-up step was then carried out with 2 × 200 μl 70% ethanol washes, and the end-prepped DNA was eluted in nuclease-free water preheated to 55°C. Adapter ligation was then carried out in a 50 μl reaction volume with 30 μl DNA, 12.5 μl ligation buffer (LNB), 5 μl T4 DNA ligase, and 2.5 μl adapter mix (AMX). The reaction was incubated for 10 min at room temperature before a 0.4 × AMPure XP bead clean-up and 2 × 125 μl long fragment buffer (LFB) washes (pellets were resuspended when washed). DNA was eluted in 7 μl elution buffer (EB) and incubated for 5 min at 37°C. One μl eluted DNA was quantified using a Qubit 4 fluorometer (Invitrogen) and the 1 × dsDNA HS assay. Between 25 and 50 ng of adapter-ligated DNA was loaded onto the FLO-FLG001 Flongle flow cell (R9.4.1; ONT). Sequencing was run for 24 h and fast5 output files were basecalled using the high-accuracy model (HAC) of the Guppy v3.4.5 (ONT) basecaller.

### DNA sequence processing and genome assembly

Sequencing read quality profiles were examined using FastQC v0.11.9 [44] and used to choose filtering and trimming parameters. Reads were filtered according to their quality scores (minimum phred score = 11) and length (minimum length = 350 bp)**;** the first 50 bp were trimmed from the start of the reads using Nanofilt v2.8.0 [45]. Filtered and trimmed reads were checked using FastQC before being taken forward for genome assembly. Genomes were assembled using Flye v2.9-b1768 [46] specifying an input genome size of 200 Kbp and using the longest reads with 50X coverage for initial disjointing assembly. The resulting assemblies were polished with the filtered reads using Medaka v1.2.1 (ONT, Oxford, UK) and the r941_min_high_g360 configuration file. Genome annotation was done with Prokka v1.14.6 [47] and the —kingdom Viruses parameter. The Prokka output files of predicted protein sequences were used for functional prediction using InterproScan v5.57–90 [48, 49].

### Phylogenetic reconstruction

The phylogeny of Phycodnaviridae infecting green algae was based on the viral 22 core proteins (Fig. S3A) shared among the chloroviruses and prasinoviruses [50], and on the protein sequence of the viral DNA polymerase B (Fig. S3B). For the shared protein set, the predicted protein sequences of MpoV-45T and 46T were searched using homolog sequences from genome-sequenced *Micromonas* viruses as BLASTP search queries. The identified MpoV core proteins were then aligned to the original core protein alignment using MAFFT v7 (—add and G-INS-i all-pair global alignment options [51], and the alignment was subsequently edited with trimAL [52] using the gap distribution algorithm. We used the same alignment methodology for the DNA polymerase B protein sequences. The shared protein and DNA polymerase B phylogenetic trees was reconstructed with IQTREE v2.2.2.6 [53] using the substitution model best suiting the data; branch support values were based on 1000 non-parametric bootstraps (Fig. S3). Orthologous proteins between MpoV-45T and MpoV-46T were identified by running OrthoFinder v2.5.5 [54] on prasinovirus proteomes (using all available predicted proteomes) with Diamond [55] in ultra-sensitive mode, and the pairwise ortholog table for MpoV-45T and MpoV-46T were extracted from the output.

### RNA extraction

For transcriptomics analysis, a similar experimental set-up was used as for the competition experiments, but with larger volumes (250 ml) and host abundances of 2x10^6^ cells ml^−1^ (v:a still 10) to ensure sufficient RNA yields. Samples of 30 ml were taken within half an hour after virus addition (T0) and 6, 12, 18, 24, and 30 h p.i. (T6, T12, T18, T24, T30), after which they were centrifuged at 4000 × g for 30 min at 4°C. The supernatant was discarded, and the remaining pellet was gently resuspended in 100 μl of the remaining liquid and transferred to 2 ml microcentrifuge tubes. After another centrifugation step at 10 000 × *g* for 5 min at 4°C the supernatant was carefully removed (using a pipette) and samples were snap frozen in liquid nitrogen. Sample handling was randomized and total handling time (including centrifugation) for all samples was under 1.5 h (except for T0 it was 2 h). Samples were stored at −80°C until further analysis. For MpoV-45T the T30 samples failed because the RNA yield was too low.

Total RNA was extracted from cell pellets using the Qiagen RNeasy Plant Mini Kit (Qiagen, Hilden, Germany) following the manufacturer’s instructions. Cell lysis was carried out by adding approximately 250 mg of previously autoclaved 0.5 mm glass beads to the pellets and 450 μl Buffer RLT, followed by vigorous vortexing for ~1 min. The lysate was pipetted into a QIAshredder homogenizer spin column, omitting the beads from the previous step, and centrifuged for 2 min at full speed (20 000 × *g*). The flow through was carefully transferred to a 1.5 ml microcentrifuge tube. 0.5 volumes of 100% ethanol were added to the cleared lysate and immediately mixed by gentle pipetting, before transferring the mixture to an RNeasy mini spin column and centrifuging for 15 s at 8000 × *g*. The flow through was discarded and 700 μl buffer RW1 were added to the spin column, followed by centrifugation for 15 s at 8000 × g. The flow through was again discarded and 500 μl buffer RPE were added to the spin column, followed by a centrifugation for 15 s at 8000 × *g* and discarding the flow through. This step was repeated once for a total of two washes with buffer RPE. The spin column was then dry centrifuged for 1 min at 20 000 × g to remove residual buffer from the membrane. To elute the RNA from the spin column membrane, the column was placed in a clean 1.5 ml microcentrifuge tube and 60 μl RNase-free water was added directly to the spin column membrane and centrifuged for 1 min at 8000 × *g*. The eluate was collected from the microcentrifuge tube and pipetted back onto the spin column membrane for a second elution step to increase the RNA yield/concentration. One μl of each extract was quantified by Qubit fluorometer (Invitrogen) using the RNA HS assay, and RNA quality was assessed by Agilent TapeStation 4200 using the RNA HS assay. Samples with sufficient RNA yield and quality (N = 60) were brought forward for sequencing library preparation.

### RNA library prep and sequencing

PolyA-tailed RNA (mRNA) was isolated from total RNA using the NEB PolyA mRNA Magnetic Isolation Module (E7490L) following the manufacturer’s instructions. RNA libraries were prepared for Illumina sequencing using the NEB Ultra II directional RNA Prep Kit (E7760L) and NEB Multiplex oligos UDI UMI RNA Set 1 (E7416S) following the manufacturer’s instructions. Prepared libraries were checked by Qubit fluorometer using the 1x dsDNA HS assay and TapeStation D1000 assay to ensure sufficient library yield and quality and to enable equimolar pooling for sequencing. Three pools of different concentrations were made up: pool 1 comprised 34 libraries at 5 nM, pool 2 comprised 16 libraries at 1 nM, and pool 3 comprised 4 libraries at 0.1 nM (54 libraries in total were sequenced). If required, libraries were diluted in low EDTA TE buffer (10 mM Tris/0.1 mM EDTA). Each pool then underwent a minimum of two AMPure XP bead clean up steps to remove short sequences and primer dimer, with pool 3 requiring a third clean up step. Briefly, pools were made up to a minimum volume of 50 μl and 0.8 v/v AMPure XP beads were added, followed by two washes with 80% ethanol (200 μl) and elution in low ETDA TE buffer. The 150 bp paired-end libraries were sequenced on a NovaSeq platform (Illumina, San Diego, USA).

### RNA sequence pre-processing and transcriptome analysis

Fastp v0.23.4 [56] (minimum length required 50) was used to remove adapters and trim low-quality reads from raw sequence data. Quality control reports were compiled using FastQC v0.12.1 [44], MultiQC v1.14 [57], and fastp. SortMERNA v4.3.6 [58] was used to remove any reads aligning to rRNA sequences in the SILVA 138.1 database. The remaining reads were aligned and quantified against the MpoV-45T and MpoV-46T genomes using Star v2.7.10b [59] with the default parameters. DESeq2 v3.16 [60] was used to quantify and normalize the log_2_ fold changes in transcript abundances for each gene. Standardization was carried out by dividing the normalized counts by gene length. Non-negative matrix factorization (NMF) was used to verify that timepoint replicates were comparable so that gene expression values for replicates could be averaged for each timepoint (Fig. S4). Samples were partitioned using the R package NMF v0.30.4.900 [61]. NMF decomposes the gene abundance matrix into the product of 2 matrices. The coefficient matrix describes the overall structure of the gene abundance matrix. The optimal results were obtained for the nsNMF algorithm, random seed of the factorized matrices, and a rank value of 3. We performed the final analysis with rank of 3 for MpoV-45T and a rank of 4 for MpoV-46T, with random seed and the nsNMF algorithm. Mean gene expression values per timepoint were centered, scaled, and plotted as heatmaps using the ComplexHeatmap v2.16.0 R package [62].

## Results

### Dual infection dynamics

**Figure 1:**
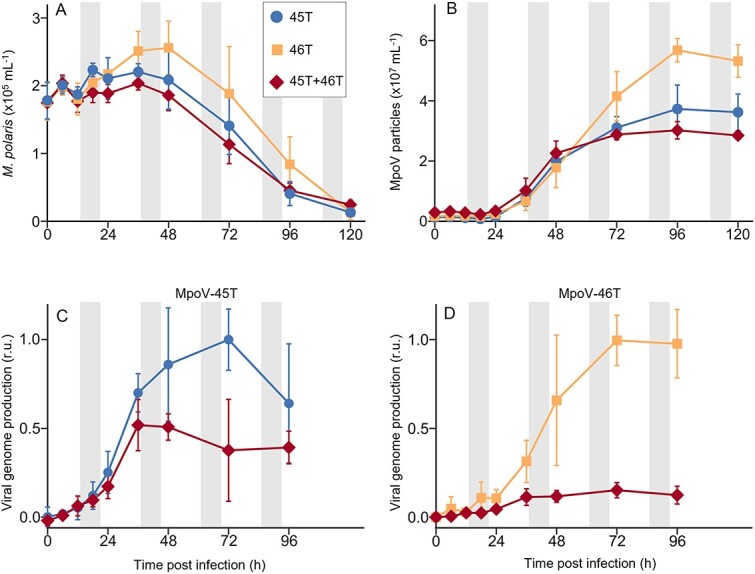
Dual infection dynamics of *Micromonas polaris* with its viruses MpoV-45T and MpoV-46T, compared to single virus infection. (**A**), *Micromonas polaris* abundances and (**B**), MpoV particle dynamics obtained by flow cytometry. (**C**, **D**) viral genome production of MpoV-45T (**C**) and MpoV-46T (**D**). Viral genome abundances are min-max normalized with the respective single virus infection treatment as reference. Grey shaded areas indicate dark periods. Data show mean ± SD (with N = 6), based on two independent experiments with three replicates each, illustrating good reproducibility (see Fig. S1 for the infection dynamics per individual experiment).

We performed dual infection experiments at 3°C, inoculating the host, *Micromonas polaris* strain RCC2258, either with MpoV-45T or MpoV-46T individually or both simultaneously. Flow cytometry showed that both viruses MpoV-45T and MpoV-46T displayed similar infection cycle dynamics (Fig. 1). Algal host cell lysis started 36–48 h post infection (p.i.) (Fig. 1A) and the MpoV latent periods were 18–24 h (Fig. 1B, S5). The viral particle burst size was significantly higher for MpoV-46T compared to MpoV-45T (226 ± 31 vs 172 ± 19 viruses per lysed host cell; Student’s t-test, t_10_ = −3.6, *P* = 0.005). Dual infection of *M. polaris* with MpoV-45T and MpoV-46T did not affect host lysis dynamics (Fig. 1A, B) but did cause a significant reduction in particle burst size compared to single infection by either of the viruses (151 ± 16 viruses per lysed cell; Student’s t-test, t_16_ = −2.0, *P* = 0.009).

Because MpoV-45T and MpoV-46T cannot be discriminated by flow cytometry, we performed qPCR targeting the viral DNA polymerase gene B (*polB*) to examine the infection dynamics of each virus in the dual infection experiments (Fig. 1C, D). Genome production (represented by *polB*, considering there is one copy of the DNA polymerase gene per viral genome) started ~6 h earlier for MpoV-45T than for MpoV-46T (6–12 h p.i. vs 12–18 h p.i.). Similarly, maximum genome production of MpoV-45T was reached earlier than that of MpoV-46T (at 48 h vs 72 h p.i.). The maximum genome production rates for MpoV-45T and MpoV-46T single infection treatments were comparable (i.e., 0.029 ± 0.007 and 0.027 ± 0.013 h^−1^, respectively). The dual infection treatment, however, resulted in a strongly reduced genome production for both viruses compared to the single treatments (Fig. 1C, D); viral genome burst size of MpoV-46T was only 21 ± 4% of the single treatments (Student’s t-tests on the qPCR results of the two independent experiments: t_4_ = 7.8 and 10.2, *P* = 0.001 and 0.001) and for MpoV-45T it was 65 ± 8% (Student’s t-tests: t_4_ = 4.5 and 10.2, *P* = 0.011 and 0.001).

### Viral genomes and phylogenetics

**Figure 2:**
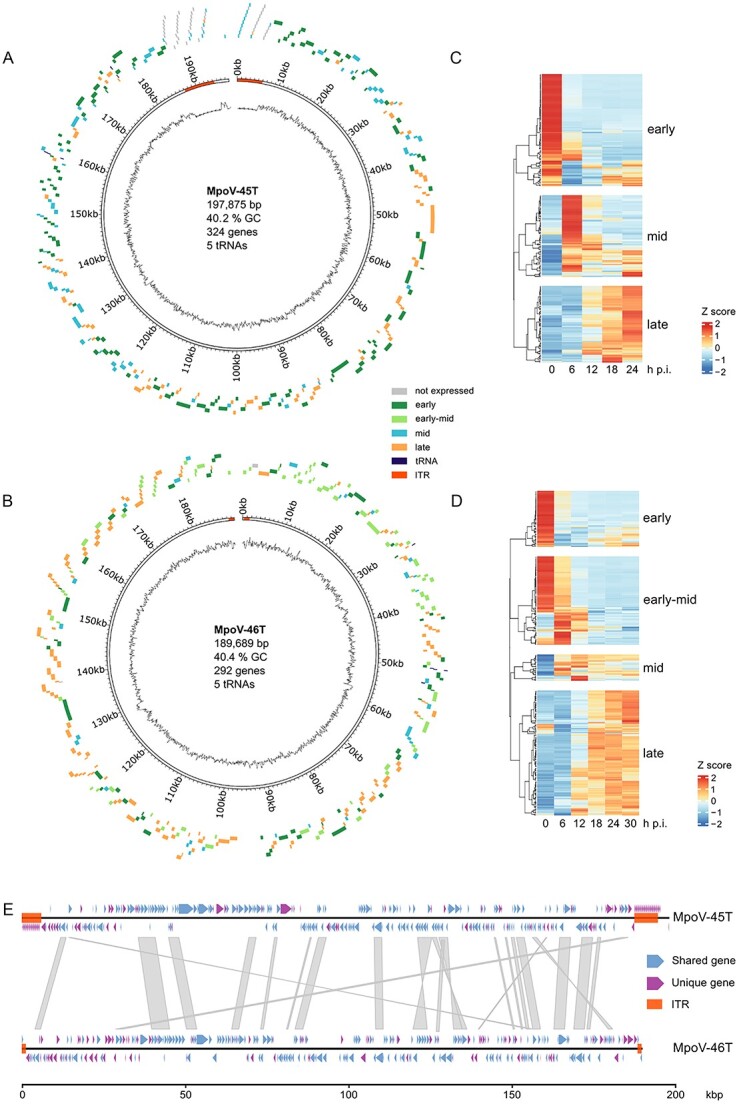
Genomes of MpoV-45T and MpoV-46T. (**A**, **B**) circular representation [78] of the MpoV-45T genome (**A**) and MpoV-46T genome (**B**), with gene loci represented as blocks outside of the circle and colored according to their kinetic class in single infection. The line at the centre of the circle represents the GC content throughout the genome (window size = 300 bp). Segments at either ends of the genome indicate the ITR positions. Genome metrics are given at the centre of the circle. (**C**, **D**) gene expression heatmap, made using ComplexHeatmap [62] in R, showing kinetic classes of MpoV-45T (**C**) and MpoV-46T (**D**) in the single-infection treatments. Expression values have been averaged across timepoint replicates, centered and scaled. (**E**) genome synteny between MpoV-45T and MpoV-46T, made using gggenomes [79] in R. Red blocks indicate ITR positions, and colored arrows show gene loci and whether an ortholog of the gene exists in the other virus or not. Grey blocks between the genomes denote areas of DNA sequence homology as determined by a Minimap2 [80] alignment with the parameters -X,-N 50 and -p 0.1.

To obtain a better understanding of the similarities and differences between the two viruses, and to enable transcriptomics read mapping to viral genomes, we sequenced and assembled the genomes of both MpoVs. The genome size of MpoV-45T and MpoV-46T (197.9 and 189.7 kb) and GC content (40.2 and 40.4%, respectively) were comparable (Fig. 2A, B). Although both genomes possessed inverted terminal repeats (ITRs), the MpoV-45T ITRs were much longer than those of MpoV-46T. The 5’ ITR of MpoV-45T was 5959 bp in length compared to 1266 bp in MpoV-46T, and the 3’ ITR was 7166 bp in MpoV-45T as opposed to 1264 bp in MpoV-46T. The reason for the >1 kb length difference in the ITRs of MpoV-45T is unknown. The depth of genome coverage for MpoV-45T and MpoV-46T was 700x and 1000x, respectively. The good coverage in combination with the use of long read sequencing provides confidence in our MpoV genome assemblies. There was no sequence similarity between the ITRs of MpoV-45T and those of MpoV-46T. MpoV-45T had 324 predicted genes, whereas MpoV-46T had 292 predicted genes. There were 154 orthogroups shared between the two virus genomes, comprising 192 genes in MpoV-45T and 197 genes in MpoV-46T (Fig. 2E). MpoV-46T possessed all 22 of the previously identified core genes of the green algal viruses (Prasinoviruses and Chloroviruses) [50], whereas MpoV-45T lacked the core gene 2-oxoglutarate/iron (II)-dependent oxygenase. Phylogenetic reconstructions of relationships between the green algal viruses showed a paraphyletic relationship between viruses infecting *Micromonas* spp., which formed two distinct clades (Fig. S3). MpoV-45T clustered within Micromonas virus clade 1 (MV-1) alongside MpV-12T, and MpoV-46T clustered within Micromonas virus clade 2 (MV-2) along with other MpVs including RCC1109 virus 1. The DNA polymerase B-based phylogeny also supported the paraphyly among Micromonas viruses (Fig. S3).

### Viral transcriptome

**Figure 3:**
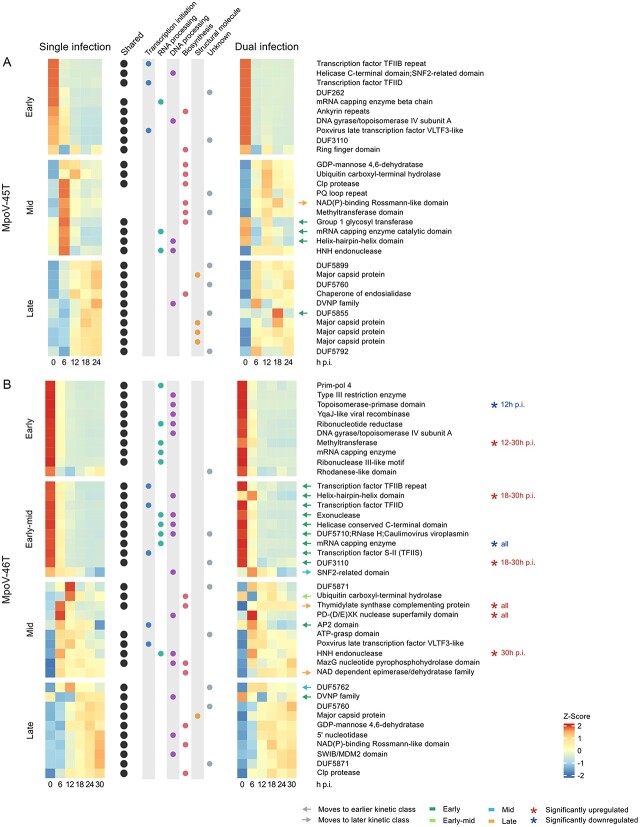
Top ten expressed and annotated genes in single and dual infection experiments in each kinetic class for MpoV-45T (**A**) and MpoV-46T (**B**). Mean expression values per timepoint are centered and scaled. Genes with orthologs in MpoV-45T are marked with a black circle. Information on gene function is given in the central columns with colored circles. Genes which switch to a different kinetic class in dual infection are indicated with arrows colored according to the kinetic class they move to. Genes that are significantly up- or downregulated in dual infection compared to single infection are denoted with asterisks.

We analysed the viral transcriptomes to assess (i) if the two MpoV viruses differed in their gene expression patterns, and (ii) if the gene expression of each virus changed in response to the presence of the other virus. Both viruses exhibited distinct temporal patterns of gene expression in the single infection treatments (Fig. 2C, D). MpoV-45T genes clustered into three kinetic classes (early, mid, and late) while MpoV-46T genes clustered into four kinetic classes (early, early-mid, mid, and late). In MpoV-45T, early genes were most highly expressed within 30 min after infection (T0), mid genes were most highly expressed at 6 and 12 h p.i., and late genes from 12 to 24 h post infection. MpoV-46T early genes were most strongly expressed at 0 h p.i., whereas early-mid genes were primarily expressed at 0 and 6 h post infection. MpoV-46T mid genes were highly expressed at 6 and 12 h p.i., with the expression of some of the genes continuing at lower levels up to 30 h p.i., and late genes were predominantly expressed from 18 to 30 h post infection. Overall, MpoV-46T late genes had lower expression levels than the other MpoV-46T kinetic classes. The most strongly expressed early genes in MpoV-46T comprised genes involved in DNA and RNA conformational change, such as DNA gyrase and DNA topoisomerases. Other MpoV-46T early gene functions related to RNA processing and repair, DNA and RNA degradation, and deoxyribonucleotide synthesis. Highly expressed genes in the MpoV-45T early and MpoV-46T early-mid classes included several transcription factors, alongside genes involved in transcription initiation and nucleic acid processing (Fig. 3). The highly expressed genes in the MpoV-45T mid class were mostly involved in biosynthesis pathways, such as GDP-mannose 4,6-dehydratase and a group 1 glycosyltransferase. In addition, most of the predicted coding regions within the MpoV-45T ITR regions were expressed in the mid kinetic class. *PolB*, although not among the most highly expressed genes, fell into the mid kinetic class in MpoV-45T. Some highly expressed genes of the MpoV-46T mid class had roles in biosynthesis, but there were also two transcription factors and genes involved in nucleic acid processing. The late class of MpoV-45T comprised four highly expressed major capsid proteins and a highly expressed dinoflagellate-viral-nucleoprotein (DVNP), involved in chromatin packaging. A DVNP and major capsid protein were also highly expressed in the MpoV-46T late class, along with highly expressed genes involved in DNA processing.

In the dual infection treatment, both viruses had genes that were significantly differentially expressed (log_2_ fold change > ±2; *P*-adj < 0.001) when compared to single infection, with MpoV-46T more strongly affected than MpoV-45T (Fig. 3, Fig. S6, S7). A total of seven MpoV-45T genes were differentially expressed in the dual infection relative to single infection with MpoV-45T: four genes were significantly upregulated at 12 h p.i. and three genes significantly upregulated at 18 h post infection. This upregulation mostly affected genes within the ITRs (Fig. S6). In contrast, MpoV-46T had 91 genes in total that were differentially expressed in dual infection; 41 of them were downregulated and 50 upregulated. MpoV-46T also displayed a distinct temporal pattern of differential gene expression (Fig. 3B, S6). At 6 h p.i., many genes were up- or downregulated in dual infection, followed by a strong pattern of upregulation for most differentially expressed genes during from 12–30 h p.i., including some of the most expressed genes of the four kinetic classes (Fig. 3B). Among the upregulated genes of MpoV-46T in dual infection were a thymidylate synthase (*thyX*) which is part of the pyrimidine deoxyribonucleoside biosynthesis pathway that produces deoxythymidine monophosphate (dTMP) [63–65]. Additionally, several genes related to DNA modification and repair, including multiple nucleases, were upregulated in MpoV-46T. Downregulated genes for MpoV-46T in dual infection included a topoisomerase-primase domain and mRNA capping enzyme (Fig. 3B).

### Delayed dual infection

**Figure 4:**
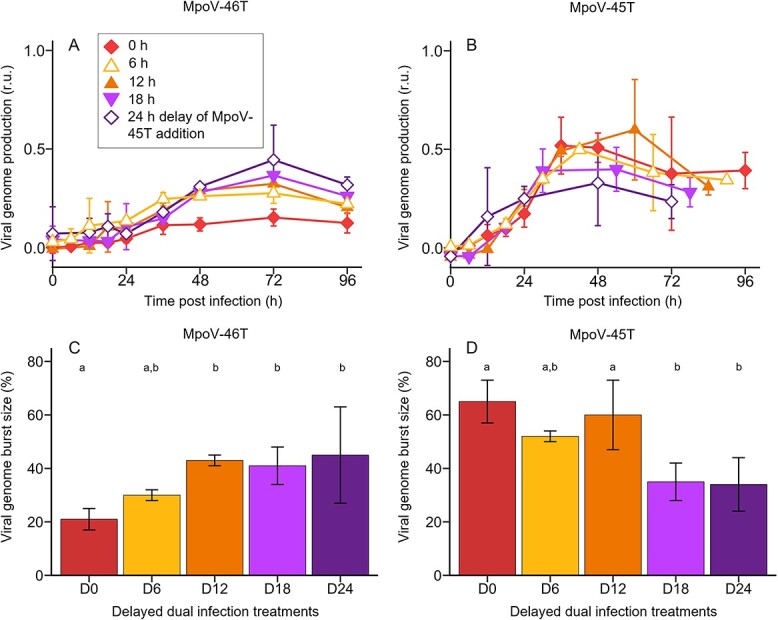
Viral genome production and burst size for the delay treatments, in which MpoV-45T is added to a culture infected with MpoV-46T at the same time (D0) and 6, 12, 18, and 24 h later (D6, D12, D18, D24). (**A**, **B**) temporal dynamics of viral genome production of MpoV-46T (**A**) and MpoV-45T (**B**), in dual infection treatments in which MpoV-45T is added 0, 6, 12, 18, and 24 h after infection with MpoV-46T. The virus growth curves all start the moment of infection. For the delayed infection with MpoV-45T (B) this means that the delay time is subtracted. Viral genome production is min-max normalized with the respective single virus infection treatment as reference. (**C**, **D**) viral genome burst size (defined as the number of genome copies produced per lysed host cell) in the delayed dual infection treatments relative to the single infection treatment (i.e., expressed as percentage of single infection), for MpoV-46T (**C**) and MpoV-45T (**D**). Results are shown as averages ± standard derivation (N = 3, except for D0 where N = 6). Bars labelled with different letters indicate significant differences, as tested by a one-way ANOVA followed by multiple comparison of means using Tukey’s HSD test (with family-wise error rate of <0.05).

Because MpoV-45T initiated its genome production earlier than MpoV-46T, and its genome production was much less reduced in the dual infection treatment (relative to single infection) (Fig. 1C, D), we postulated that the timing of virus inoculation could play a role in shaping the outcomes in virus–virus competitive interactions. To investigate this, we delayed infection with MpoV-45T for 6, 12, 18, and 24 h after first infecting the host with MpoV-46T (the single MpoV-45T infection controls were not affected by different starting times during the diel cycle; Fig. S8). The delayed additions of MpoV-45T did not significantly affect overall host lysis dynamics, viral latent period and particle burst size when compared to the non-delayed dual treatment (Fig. S9). It did result in a 2-fold higher MpoV-46T genome production in the 12–24 h delay treatments than in the non-delayed dual treatment (one-way ANOVA: *F*_4,18_ = 7.3, *P* = 0.00025) (Fig. 4A, C). Still, the viral genome burst sizes in these delayed dual infection treatments were significantly reduced compared to the single infection treatment (55–59% lower, ANOVA: *F*_4,10_ = 24.7, *P* = 0.0004).

Increasing the delay in adding MpoV-45T to MpoV-46T did not affect the MpoV-45T genome production rate (i.e., the slope in Fig. 4B), but its genome production did level off earlier with increasing delay (Fig. 4B). MpoV-45T viral genome burst size (Fig. 4D) became significantly reduced compared to the non-delayed treatment only for the 18 h and 24 h delay treatments (one-way ANOVA: *F*_4,18_ = 10.5, *P* = 0.00051), diminishing from 65 ± 8% to 34 ± 10% of the single-infection treatment (Fig. 4D). Despite its strongly delayed infection, the MpoV-45T genome burst sizes after 18 h and 24 h delay were still considerable.

### Temperature effect on virus competition dynamics

**Figure 5:**
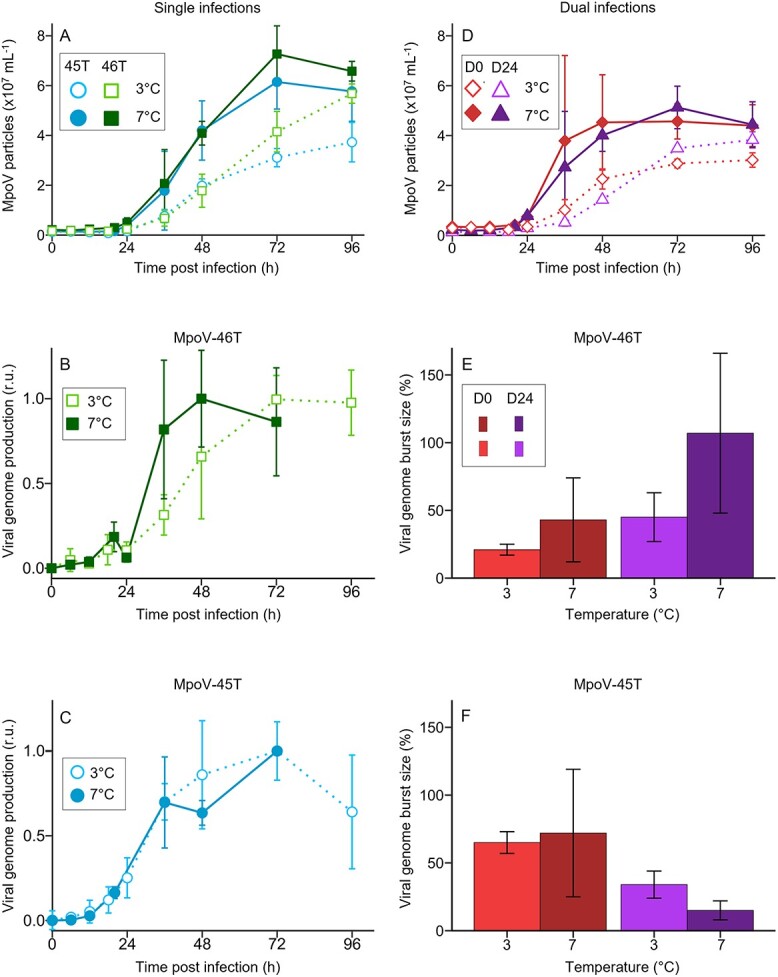
Comparison of the infection dynamics at two different temperatures (3°C and 7°C). (**A**) MpoV particle abundances during single infection at 3°C (dotted line, open symbol) vs 7°C (solid line, closed symbol). (**B**) MpoV-46T viral genome production at 3°C vs 7°C. (**C**) MpoV-45T viral genome production at 3°C vs 7°C. Viral genome production is min-max normalized with the respective single virus infection treatment as reference. (**D**) MpoV particle abundances at 3°C vs 7°C during dual infection without delay (D0) and with 24 h delayed (D24) addition of MpoV-45T. (**E**, **F**) viral genome burst size (defined as the number of genome copies produced per lysed host cell; and expressed as percentage of single infection) of MpoV-46T (**E**) and MpoV-45T (**F**) at 3°C and 7°C during dual infection without delay (D0) and with 24 h delayed (D24) addition of MpoV-45T. Results are shown as mean ± SD (N = 3 except for 3°C single and non-delayed dual treatment where N = 6).

In addition to the experiments at 3°C, we also performed infection experiments at 7°C to test if temperature influenced virus infection dynamics and competition outcome (Fig. 5). The higher temperature resulted in faster host growth of the non-infected control (0.56 ± 0.06 d^−1^ at 7°C *vs* 0.34 ± 0.04 d^−1^ at 3°C; two-sample Student’s t-test: t_7_ = −6.5, *P* = 0.0003) (Fig. S10) and shorter viral latent periods (Fig. 5A) in the single-virus infection treatments (i.e., 12–18 h for MpoV-45T and 6–12 h for MpoV-46T, compared to 18–24 h at 3°C for both viruses). Moreover, for both viruses, the rate of virus particle production at 7°C was 1.5-fold faster than at 3°C (1.36 ± 0.21 × 10^6^ vs 0.91 ± 0.14 × 10^6^ particles ml^−1^ h^−1^; Student’s t-test, t_16_ = −5.4, *P* = 0.00006; Fig. 5A) and levelled off 24 h earlier (at 72 h; Fig. 5A, D). MpoV-46T genome production was faster at 7°C than at 3°C and already reached its maximum yield at 48 h p.i. at 7°C compared to 72 h p.i. at 3°C (Fig. 5B), while MpoV-45T genome production dynamics were not affected by temperature (Fig. 5C).

Dual infection at 7°C resulted in reduced total virus particle burst sizes, reaching 72 ± 60% for the non-delayed and 62 ± 13% for the 24 h delayed dual treatment compared to the single-infection treatments at 7°C (Fig. 5D, compared with Fig. 5A). The MpoV-46T genome burst size increased strongly in the 24 h delayed dual infection at 7°C and, in contrast to 3°C, it reached a comparable burst size as the single MpoV-46T infection (Fig. 5E). Conversely, the MpoV-45T genome burst size in the 24 h delay dual infection was suppressed more strongly at 7°C than at 3°C, to only 15 ± 7% of the single-infection treatment (Fig. 5F).

## Discussion

**Figure 6:**
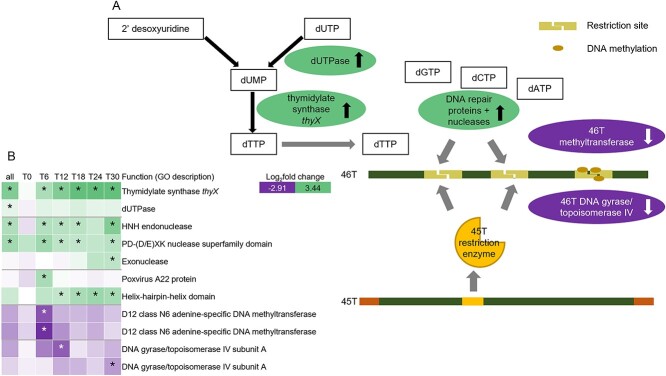
(**A**) transcriptome results pointing at possible molecular mechanism for dominant interference of MpoV-45T during coinfection with MpoV-46T. MpoV-46T genes with higher expression in dual infection compared to single infection = green, upward arrow. Lower expression in dual infection = purple, downward arrow. (**B**) Heatmap of genes involved in the proposed mechanism. Log_2_fold change refers to change in gene expression of MpoV-46T in dual infection compared to single infection. Asterisk indicates significance (*P* < 0.001).

Dual infection of two dsDNA viruses (from different prasinovirus sub-clades but with similar growth dynamics) in populations of the single-celled eukaryote *M. polaris* led to mutual interference between the viruses, in line with hypothesis H2. Both viruses showed reduced virus proliferation in the dual infection compared to the single-infection treatments, with MpoV-45T dominating MpoV-46T. The total amount of progeny viruses that the host cells could produce was limited, with dual infection producing fewer viral particles than a single infection, indicating that the viruses were competing for common substrate. The dominance of MpoV-45T indicates that, during coinfection, it was able to make more effective use of cellular resources than MpoV-46T. Both viruses share genes with a variety of different functions, with many of them being highly expressed, supporting their importance in viral replication. Certain gene products, including structural components such as capsid proteins or enzymes (e.g., replicases), may act as common goods and be shared between different viruses infecting the same cell [66]. We propose a similar mechanism for MpoV viruses related to the deoxythymidine monophosphate (dTMP) biosynthetic pathway (Fig. 6). Transcriptomic analysis revealed that MpoV-46T thymidylate synthase *thyX* and deoxyuridine triphosphate nucleotidohydrolase (dUTPase) were significantly upregulated in coinfection treatments relative to the single-infection treatment, which could point to increased competition for dTMP, an important substrate for DNA repair and replication.

 Overall, gene expression dynamics in dual infection were rapidly and significantly altered in comparison to the single-infection treatments for both viruses, indicating that the two viruses influenced each other while infecting the same host cell. In contrast, the few previous studies performed with other phytoplankton reported only mutual exclusion of coinfecting viruses [8, 9, 32], which has also been reported as a common outcome of virus competition in multicellular and bacterial hosts [5–7]. Both viruses expressed genes encoding proteins putatively involved in responses to viral interference, including restriction enzymes and other nucleases, as well as DNA methyltransferases. Restriction-modification systems have been linked to roles in both host and competitor virus genome degradation [67,68]. Several significantly upregulated MpoV-46T genes were related to nuclease activity, DNA repair, recombination, or replication, hinting at a response to DNA damage likely induced by MpoV-45T (Fig. 6). Upregulation of genes involved in dTMP synthesis could also point to an increased need for dTMP for DNA repair and replication. Additionally, several MpoV-46T genes encoding DNA methyltransferases were significantly downregulated, suggesting that MpoV-46T’s defensive response may have been impaired through a reduced capacity to protect its DNA by methylation (Fig. 6). Homologs of these genes in MpoV-45T were not significantly differentially expressed. We therefore speculate that MpoV-45T’s dominance could result from MpoV-45T-encoded restriction enzymes degrading MpoV-46T DNA, and possibly also interfering with MpoV-46T DNA methylation (Fig. 6). The downregulation of MpoV-46T topoisomerase could further point to interference with its DNA replication or repair processes. A striking feature in the MpoV-45T genome is its long inverted terminal repeats (ITRs), which comprised many significantly upregulated genes in the dual infection treatment. ITRs in other viruses have been associated with roles in genome replication and viral packaging, as well in DNA recombination [69]. Although we were not able to assign a specific function to the predicted genes within the ITR regions, their expression pattern and absence in MpoV-46T indicates that they may have played an essential role in giving MpoV-45T a competitive advantage.

Virus interference may occur at any point in the virus infection cycle [6]. Even if we experimentally delayed coinfection of MpoV-45T by 6 or 12 h, it still resulted in dominance of MpoV-45T. Superinfection, even in unicellular hosts, is typically associated with exclusion, where the virus that was added first becomes dominant [7–9], but our study clearly shows the secondary virus not only being able to replicate but also doing better than the primary virus MpoV-46T. We term this unique response, whereby the secondary virus has a competitive edge on the primary virus, “superinfection dominance”. A delay of 12 h seemed to be a threshold that allowed MpoV-46T to produce more gene copies, yet still without affecting MpoV-45T. This fits the MpoV-46T gene expression dynamics, where most early and early-mid genes were no longer highly expressed at 12-h post infection. The detrimental effects on MpoV-46T caused by introducing MpoV-45T at or after this time point appeared greatly reduced, as many important steps in viral replication (including dTMP synthesis and DNA *polB* expression) were already well underway or completed. Further delaying MpoV-45T addition did not continue to increase MpoV-46T production but did reduce MpoV-45T production. This reduction aligns with the shift towards the expression of late genes in MpoV-46T from 18 h p.i. onwards, which seemingly allowed it to exert a more substantial negative effect on MpoV-45T. Still, MpoV-46T remained significantly reduced in all dual infection treatments at 3°C, no matter how long the addition MpoV-45T was delayed. Even when added 24 h into MpoV-46T’s infection cycle, MpoV-45T genome production was still about half of its non-delayed dual infection genome production. These striking results highlight the potential of studying intricate virus–virus interactions in eukaryotic microorganisms.

MpoV-46T was able to outcompete MpoV-45T only when a 24 h delay was combined with a higher temperature (7°C). Temperature can affect virus–host interactions [70], and the shorter latent period, higher viral particle production rate, and larger burst size for both MpoV-45T and MpoV-46T match earlier reported effects of warming on MpoV-45T [71]. Similar patterns of increasing viral kinetics with temperature were found for other marine algal virus–host systems [72], including different non-polar *Micromonas* species and their viruses [73]. *Micromonas polaris* is a cold-water phytoplankton species experiencing a wide range of temperatures throughout the year. Still, its optimum growth was found between 6°C and 8°C [33], in line with our experimental results showing a significantly higher growth rate of *M. polaris* at 7°C than at 3°C. Although temperature is known to influence host metabolism [74], the change in viral kinetics cannot be attributed to host metabolism alone, as the two viruses displayed different temperature responses. In single-infection experiments, MpoV-46T genome production was faster at 7°C than at 3°C, while MpoV-45T genome production stayed the same (Fig. 5B, C). Hence, a 24 h delay in co-infection by MpoV-45T probably provided sufficient time for MpoV-46T to safeguard its DNA replication at 7°C, explaining why MpoV-46T became a stronger competitor at this higher temperature. Accordingly, differences in viral traits, even among closely related viruses, can play a critical role in how virus–host systems respond to rising temperature.

In conclusion, although we did not definitively demonstrate the presence of both viruses inside the same host cell (which would require single-cell methods beyond the scope of this study), our results do provide a strong inference of coinfection of MpoV-45T and MpoV-46T. Our findings demonstrate mutual interference between the two viruses infecting an ecologically important eukaryotic microorganism, show major changes in gene expression patterns of both viruses in the dual infection experiments, provide a molecular mechanism underlying the observed virus interference, and reveal how variation in viral traits and environmental factors control competition between the viruses. Coinfection has, to date, not been considered as a regulator of virus diversity in the ocean. However, coinfection can be expected to occur at high incidence in the marine environments [75], and interfering virus–virus interactions, as revealed in the current study, are likely to be important in directing virus diversity and evolution.

Our findings highlight the importance of temperature in regulating virus competition, suggesting shifts in virus composition under future climatic conditions. Specifically, higher summer in the Arctic will benefit MpoV-46T more than MpoV-45T. Since MpoV-46T tends to have a higher viral particle burst size than MpoV-45T, this may enhance the spread of viral infections in the *Micromonas* populations, one of the dominant phytoplankton taxa of the Arctic Ocean [33–35]. Over the last decades, the Arctic has been warming up nearly four times faster than the rest of the planet [76], and sea surface temperatures of the Arctic Ocean even reached 6 to 12°C in the summer of 2022 [77]. Our results indicate that such increased temperatures may cause major shifts in the phytoplankton virus – host systems of the Arctic Ocean, with potential cascading effects throughout the entire food web.

## Supplementary Material

Meyer_et_al_Supplement_Figures-wrae161

## Data Availability

All flow cytometry and qPCR data used in this publication, and the supplementary figures are publicly available at https://doi.org/10.25850/nioz/7b.b.ch The MpoV genomic sequencing reads are available in the Zenodo repository (https://doi.org/10.5281/zenodo.11067727) and MpoV genome assemblies are available in NCBI GenBank (accession numbers PP728250 & PP728251). Transcriptomic reads are available in the NCBI Sequence Read Archive under BioProject PRJNA1103085 (accession numbers SRR28814982-SRR28815025), and the normalized gene expression tables in Zenodo (https://doi.org/10.5281/zenodo.12686574).
