# Supplementary material for: Temperature modulates dominance of a superinfecting Arctic virus in its unicellular algal host
Source: ISME J. 2024 Aug 22;18(1):wrae161. doi: 10.1093/ismejo/wrae161 (PMC11370638; doi:10.1093/ismejo/wrae161)
Supplement: Meyer_et_al_Supplement_Figures-wrae161 [file meyer_et_al_supplement_figures-wrae161.pdf]

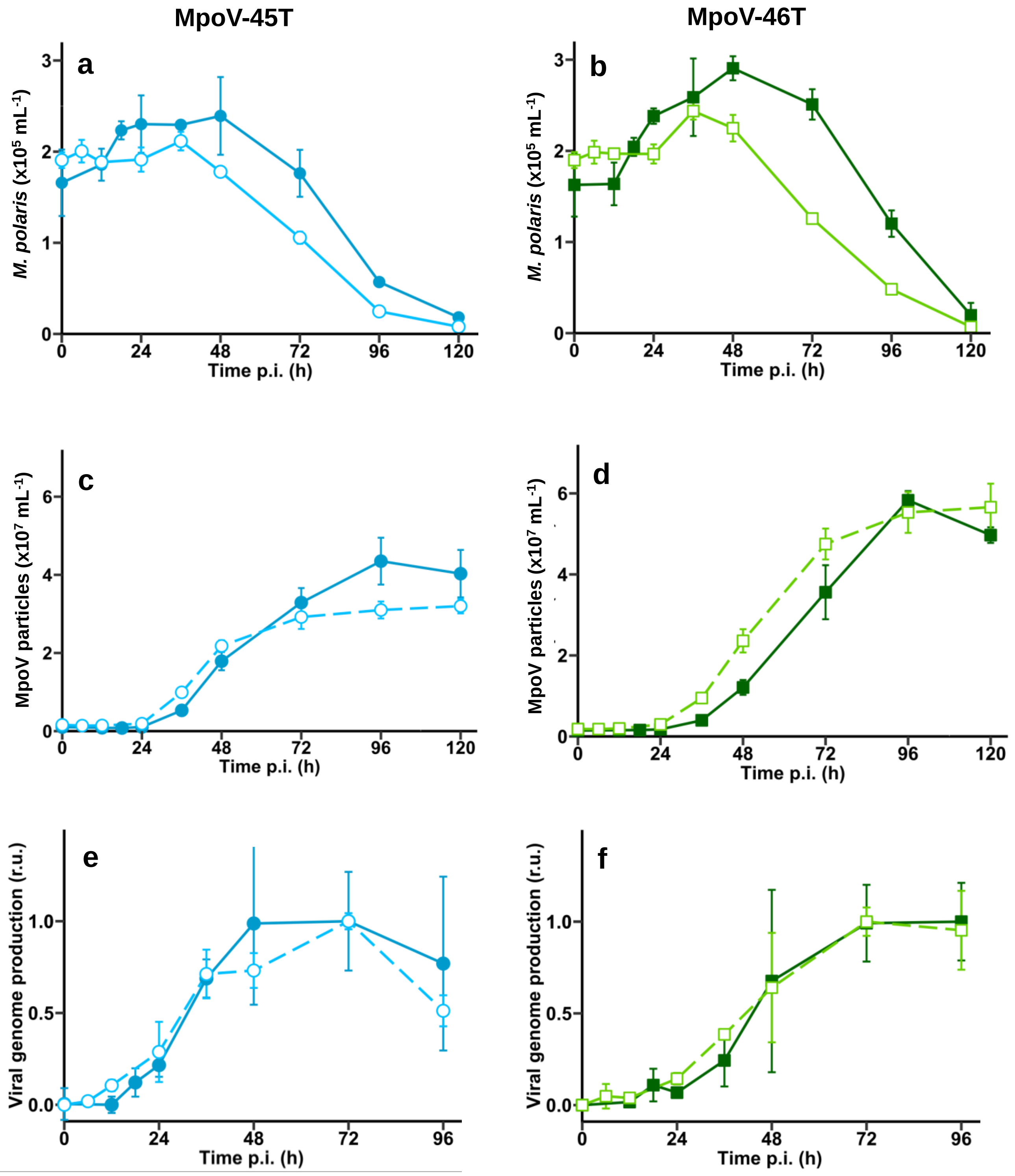

Extended Data Fig. 1. Temporal dynamics of two independent one-step growth experiments (closed and striped lines, closed and open symbols, respectively) of MpoV-45T and MpoV-46T infecting *Micromonas polaris*. **a,b,** Abundances of *M. polaris* host. **c,d,** MpoV abundances obtained by flow cytometry. **e,f,** Viral genome production over time (represented by *polB* copy abundances measured by virus-specific qPCR). **a,c,e,** Infected with MpoV-45T (blue). **b,d,f,** Infected with MpoV-46T (green). Viral genome abundances are min-max normalized with the respective single virus infection treatment as reference. Results are shown as averages ± standard derivation (n = 3); and p.i. stands for post infection.

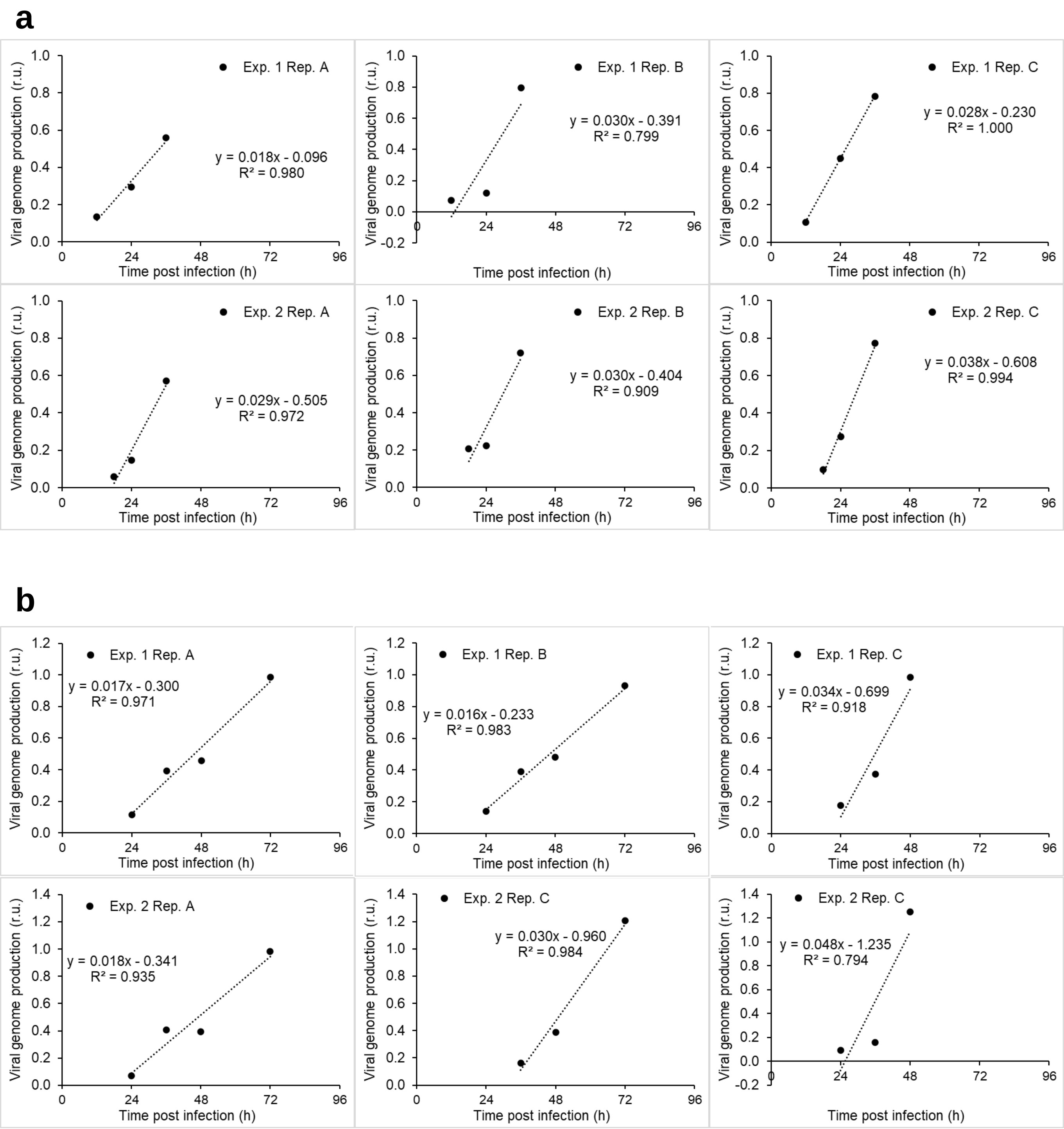

Extended Data Fig. 2. Maximum viral genome production rates (represented by *polB* copy abundances measured by virus-specific qPCR) for MpoV-45T (**a**) and MpoV-46T (**b**). Maximum viral genome production rates were calculated by fitting a linear regression over the period with the steepest slope of increase. Viral genome abundances are min-max normalized with the respective single virus infection treatment as reference. Results are from two independent experiments with three biological replicates each, data shown separate for each replicate.

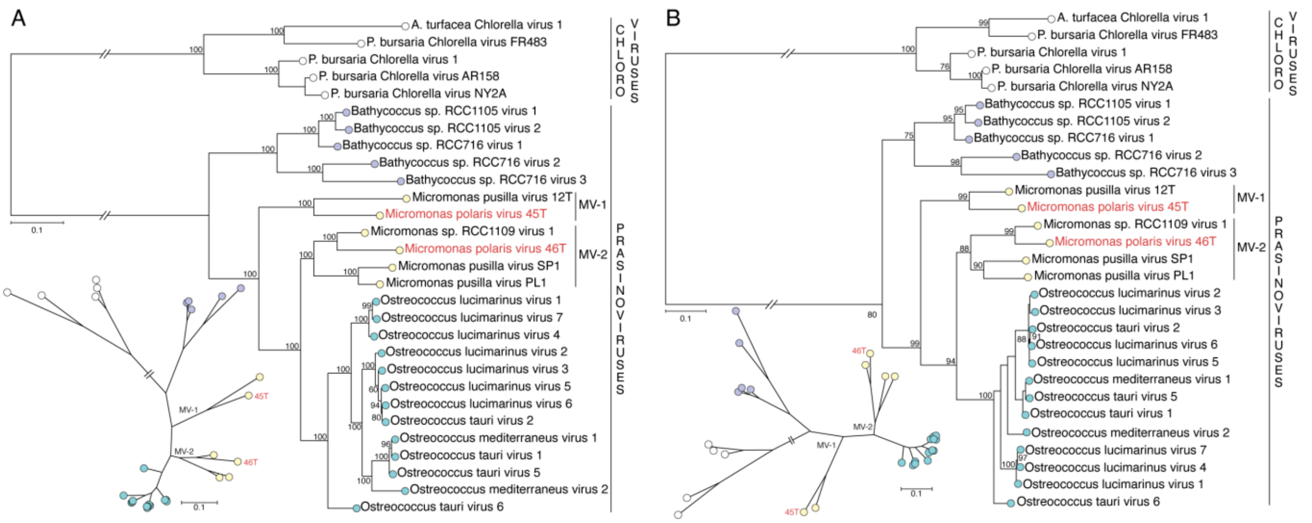

Extended Data Fig. 3. Maximum-likelihood phylogenetic trees of green algal viruses reconstructed from (**A**) a concatenated sequence alignment of 22 shared proteins (4467 parsimony-informative sites) under the WAG+F+I+R3 model and (**B**) DNA polymerase B (675 parsimony-informative sites) under the WAG+F+G4 model. The unrooted versions are shown below the midpoint-rooted trees. MpoV-45T and 46T are highlighted in red; colored circles represent the taxonomy of the viral hosts: aqua (*Ostreococcus*), purple (*Bathycoccus*), yellow (*Micromonas*), and white (chloroviruses, viruses of *Chlorella*, as outgroup). Node supports were calculated from 1000 nonparametric bootstrap replicates; only bootstrap values >75% are shown. Scale bars represent the number of estimated substitutions per site. The branches connecting the prasinoviruses to the chloroviruses were truncated for display.

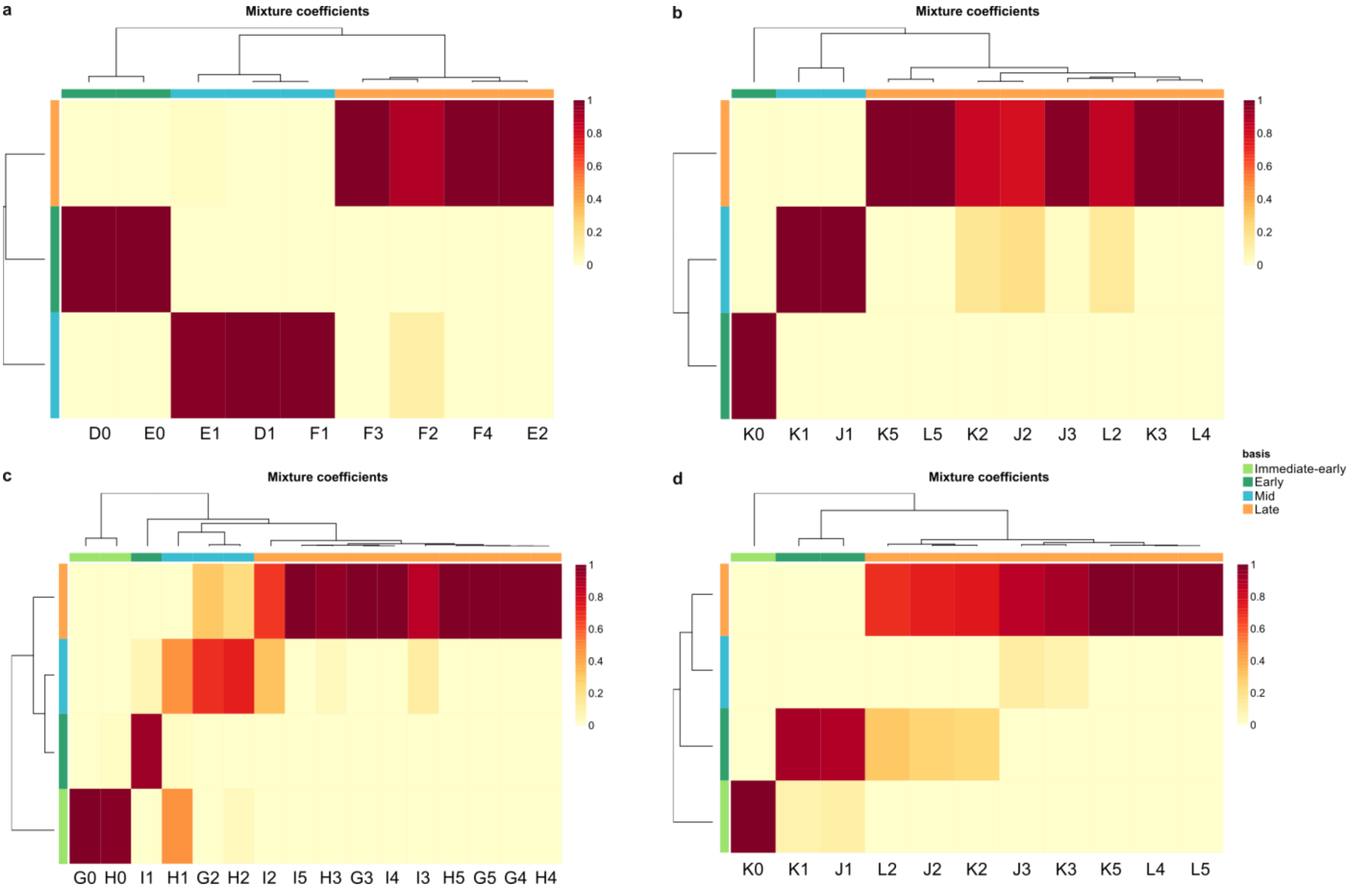

Extended Data Fig 4 NMF analysis and kinetic classes. **a-d,** heatmaps of the NMF coefficient matrix with cells coloured according to the correlation coefficients of each sample to each kinetic class for both MpoV-45T and -46T, in both single and dual infection. The matrices describe the overall structure of gene expression across the samples. Samples with the same prefix letter originated from the same replicate, and samples with the same suffix number were taken at the same timepoint (0 = 0-30 min p.i., 1 = 6h p.i., 2 = 12h p.i., 3 = 18h p.i., 4 = 24h p.i, 5 = 30h p.i.). **a**, MpoV-45T single infection. **b**, MpoV-45T dual infection. **c**, MpoV-46T single infection. **d**, MpoV-46T dual infection.

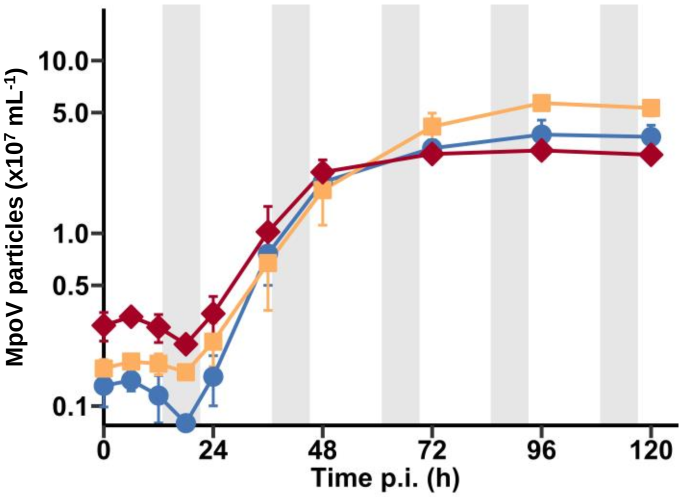

Extended Data Fig. 5. MpoV particle dynamics for single and dual infections of of *Micromonas polaris* with its viruses MpoV-45T and MpoV-46T. Grey shaded areas indicate dark periods. Data show mean ± SD (with n = 6), based on two independent experiments with three replicate measurements each.

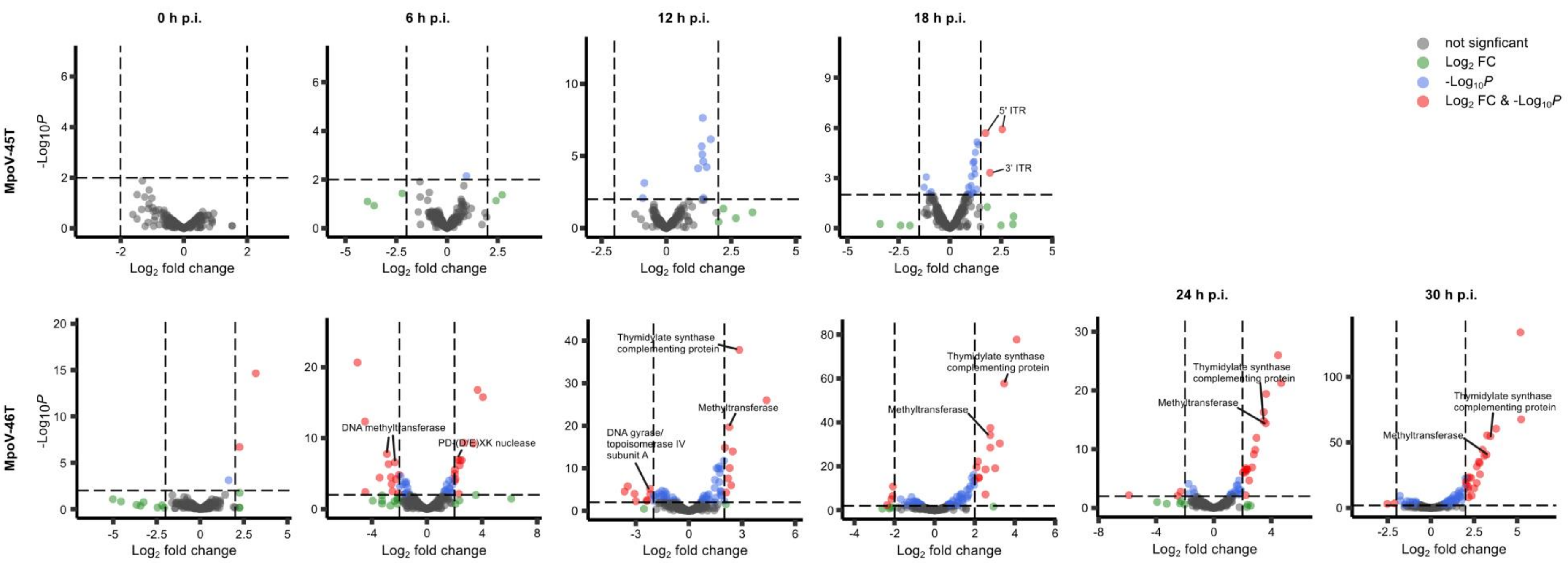

Extended Data Fig.6 : Volcano plots of differential gene expression in dual infection compared to single infection for MpoV-45T and MpoV-46T across sampling timepoints. X axes show the $\log_2$ fold change in expression levels in dual infection (significance threshold = 2); y axes show the $-\log_{10}$-transformed adjusted *p*-values of change in expression (adjusted *p*-value significance threshold = 10^{-3}). Grey points represent genes whose expression was not significantly altered between single and dual infection; green points represent genes with expression above the $\log_2$ fold change threshold in dual infection; blue points represent genes with expression above the $-\log_{10}P$ threshold and red points represent genes with expression values above both the $\log_2$ fold change and $-\log_{10}P$ thresholds. The plots in the top row show expression in MpoV-45T, and the plots in the row below show the expression in MpoV-46T. Each column is labelled with a timepoint for which the plots below show change in expression.

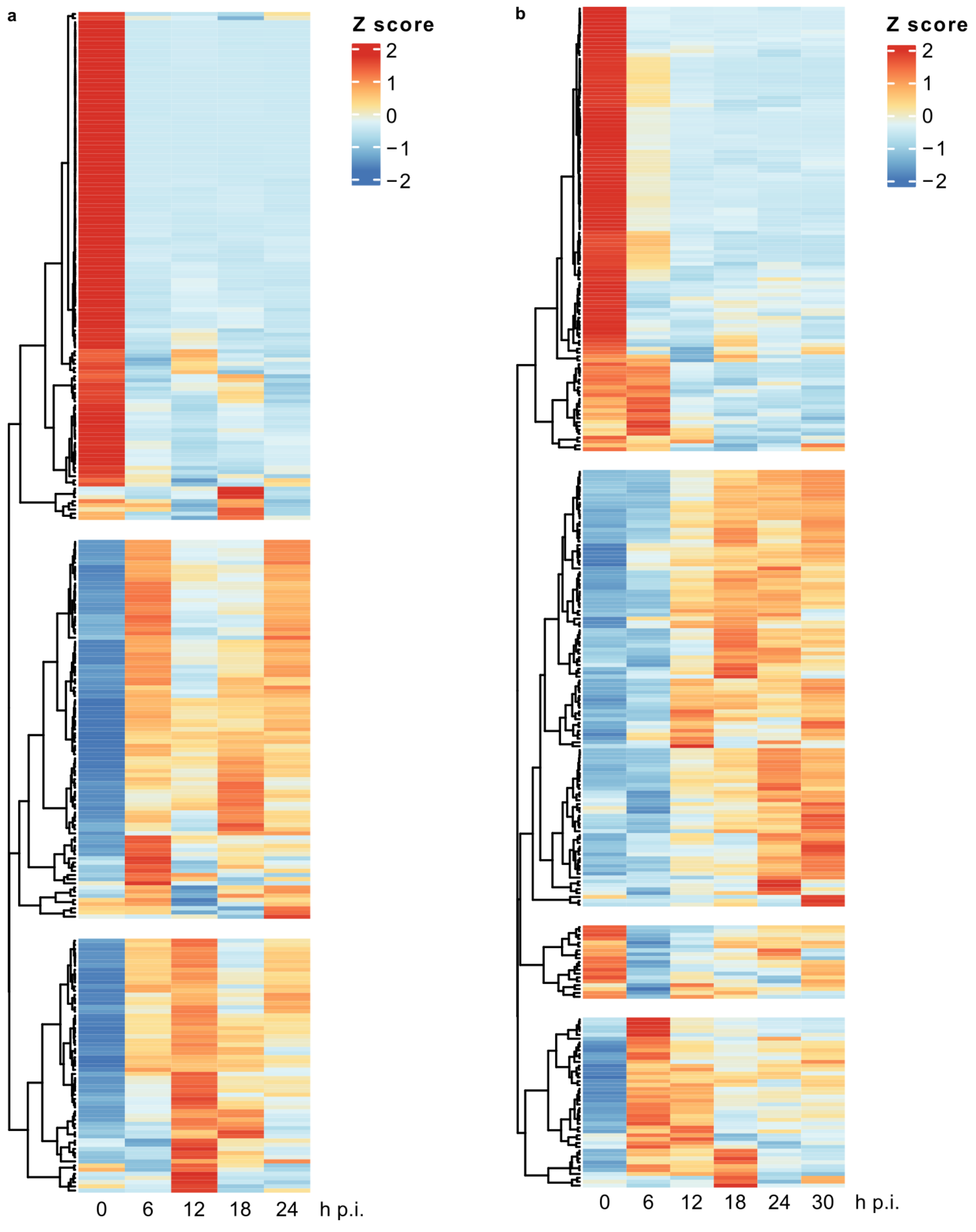

Extended Data Fig.7 Heatmaps of mean gene expression per timepoint in dual infection. Expression values have been centred and scaled (z scores). **a**, Mean expression of MpoV-45T genes per timepoint in dual infection. **b**, Mean expression of MpoV-46T genes per timepoint in dual infection.

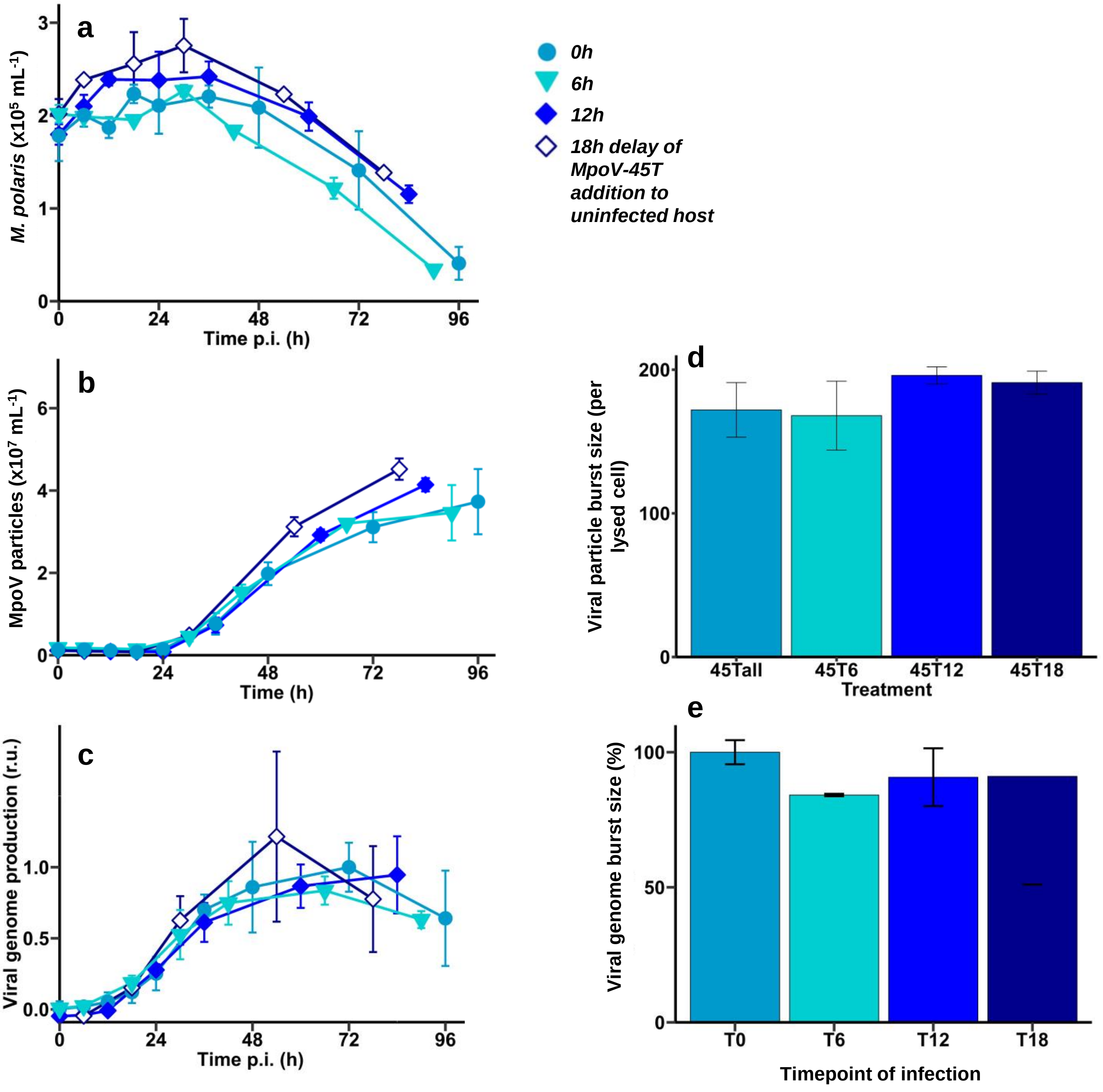

Extended Data Fig.8: MpoV-45T one-step growth experiments starting at different times of the day show consistent infection dynamics. **a,** *Micromonas polaris* host abundances. **b,** MpoV-45T particle abundances. **c,** MpoV-45T genome production (normalized to T0 single treatment). **d,** MpoV-45T particle burst size (per lysed host cell). **e,** MpoV-45T genome burst size (normalized to single infection treatment). Infection experiments were routinely performed 3h into the light cycle (16:8h L:D) (T0) and the delay treatments started 6, 12 and 18 h thereafter (T6, T12 and T18, respectively).

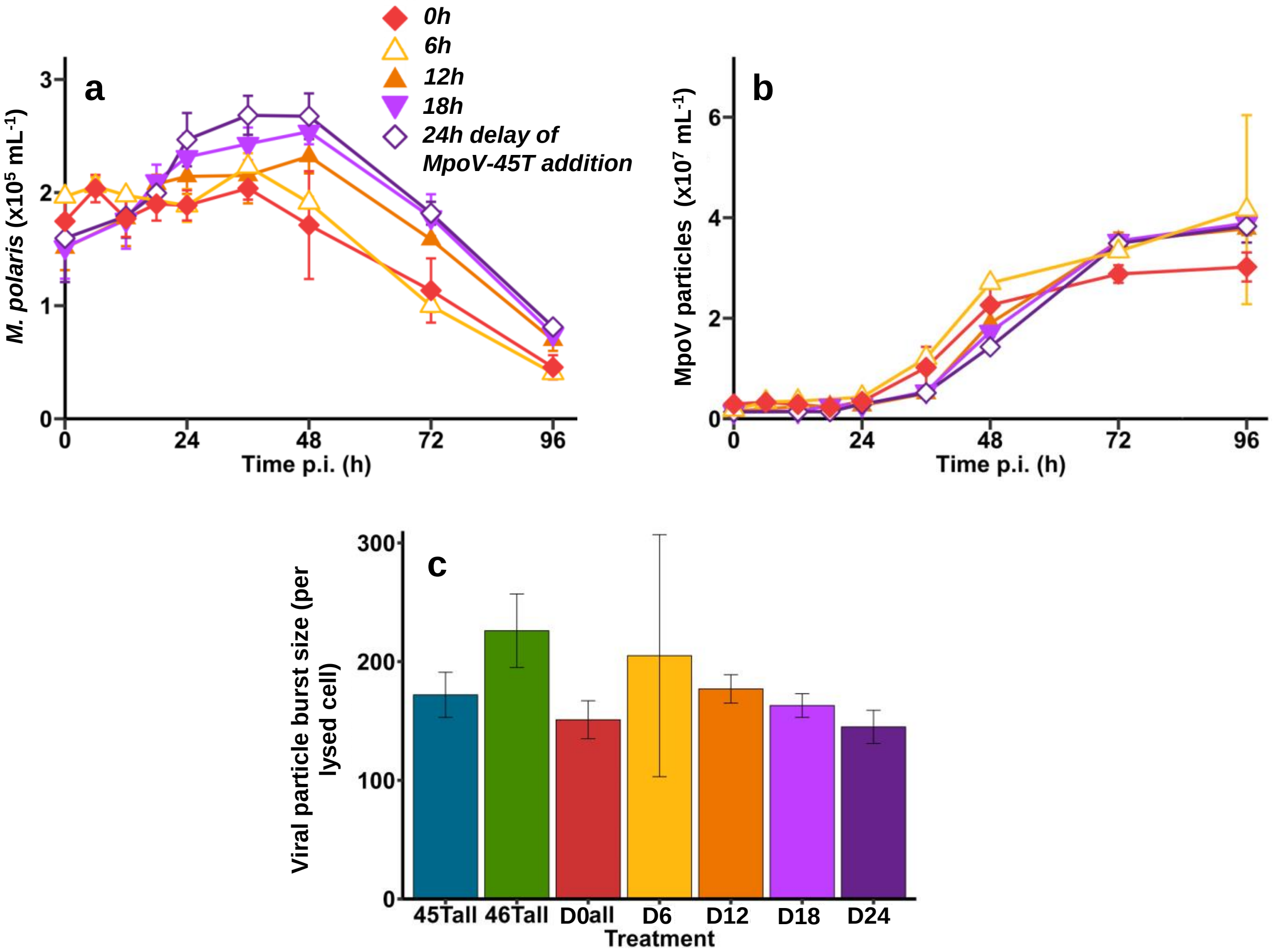

Extended Data Fig.9: FCM results for dual infection treatments, in which MpoV-45T was added to a culture infected with MpoV-46T at the same time (D0) and 6, 12, 18 and 24h later (D6, D12, D18, D24). **a,** *Micromonas polaris* host abundances. **b,** MpoV particle abundances. **c,** MpoV particle burst size (per lysed host cell) for MpoV-45T and MpoV-46T single infections, dual infection without delay (D0) and dual infections with 6, 12, 18 and 24h delay (D6, D12, D18, D24).

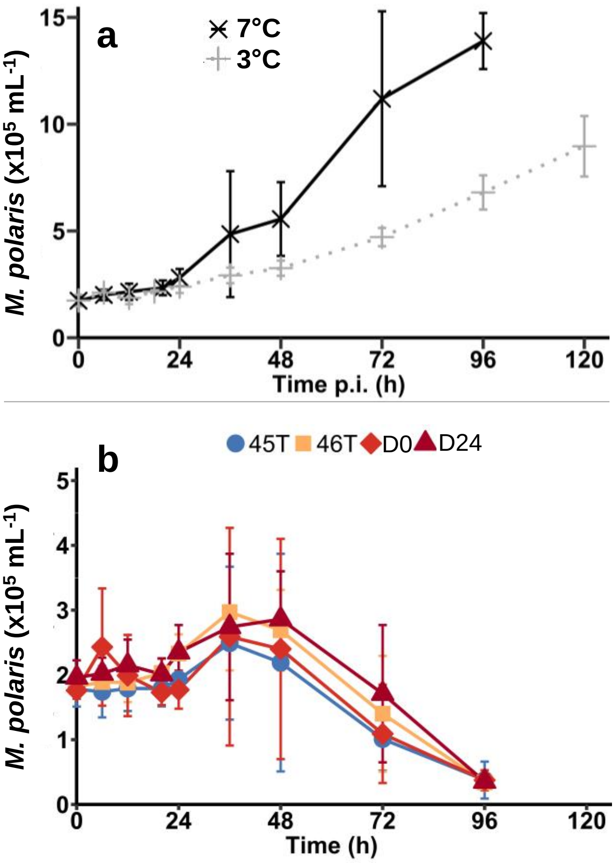

Extended Data Fig.10. Growth dynamics *M. polaris* host at different temperatures. **a,** uninfected controls at 3°C and 7°C. **b,** treatments infected with MpoV-45T and MpoV-46T at 7°C, with single infection treatments as well as dual infection treatments infected with both viruses at the same time (D0) and with a 24 h delay of adding MpoV-45T (D24).
